# Ultrasound Imaging of the Superficial Fascia in the Upper Limb: Arm and Forearm

**DOI:** 10.3390/diagnostics12081884

**Published:** 2022-08-04

**Authors:** Carmelo Pirri, Nina Pirri, Diego Guidolin, Veronica Macchi, Raffaele De Caro, Carla Stecco

**Affiliations:** 1 Department of Neurosciences, Institute of Human Anatomy, University of Padua, 35121 Padua, Italy; 2 Department of Medicine—DIMED, School of Radiology, Radiology Institute, University of Padua, 35128 Padova, Italy

**Keywords:** superficial fascia, subcutaneous tissue, ultrasonography, thickness, reliability

## Abstract

The superficial fascia has received much attention in recent years due to its important role of compartmentalizing the subcutaneous tissue. Ultrasound (US) imaging, owing to its high definition, provides the possibility of better visualizing and measuring its thickness. The aim of this study was to measure and compare, with US imaging, the thickness of superficial fascia in the arm and forearm in different regions/levels. An observational study has been performed using US imaging to measure superficial fascia thickness in the anterior and posterior regions at different levels in a sample of 30 healthy volunteers. The results for superficial fascia thickness revealed statistically significant differences (*p* < 0.0001) in the arm between the anterior and posterior regions; in terms of forearm, some statistically significant differences were found between regions/levels. However, in the posterior region/levels of the arm, the superficial fascia was thicker (0.53 ± 0.10 mm) than in the forearm (0.41 ± 0.10 mm); regarding the anterior regions/levels, the superficial fascia of the arm (0.40 ± 0.10 mm) was not statistically different than the forearm (0.40 ± 0.12 mm). In addition, the intra-rater reliability was good (ICC_2,k_: 0.88). US helps to visualize and assess the superficial fascia inside the subcutaneous tissue, improving the diagnosis of fascial dysfunction, and one of the Us parameters to reliably assess is the thickness in different regions and levels.

## 1. Introduction

The superficial fascia has received much attention in recent years due to its important role in aesthetic and reconstructive surgery and dermatology [1]. Indeed, it is a fibroelastic structure inside the subcutaneous tissue that allows for the compartmentalization of the latter in superficial adipose tissue (SAT) and deep adipose tissue (DAT) [2].

Numerous surgical approaches have been developed in which its role was highlighted, such as flaps in plastic reconstructive surgery [3,4,5] but also in abdominoplasty and liposuction in aesthetic surgery [1], leading to the need for deeper knowledge of this structure. Odobescu et al., (2021) described a novel method of pre-shaping DIEP hemi-abdominal flaps with the use of a one-step purse-string suture around the periphery of the flap, at the level of the Scarpa fascia (the superficial fascia of the abdomen), improving the projection of the flap and not putting any direct tension on the underside of the flap [5].

In fact, knowing the exact thickness of a patient’s superficial fascia reduces the risk of superficial fascia damage during a surgical procedure and makes it possible to predict the seal of the flap [2].

However, some studies have also confirmed the importance of the superficial fascia in pain [6]. Indeed, Fede et al. [7] reported that the superficial fascia of the hip region is second only to the skin in terms of innervation density. More recent studies have been carried out on this topic to understand the anatomical features of the various superficial fasciae of the body [8,9], both in cadavers and in live individuals using ultrasound (US) imaging [8,9]. The latter, owing to its high definition, provided the possibility of visualizing the musculoskeletal structures in a dynamic way, and its lower cost compared with other noninvasive methods has made it an important tool for studying fascial anatomy and pathology from a rehabilitation point of view [10].

With US, it is possible to reveal the thickening of a fascial layer and the changing of its echogenicity and then to analyze its relationships with the other anatomical structures [11]. In some studies [12,13], an increase of the US superficial fascia thickness is also related to myofascial pain.

Notably, the ultrasound data collected for the same type of fascia are affected by the ultra-sonographer, the probe position, and/or intra-individual anatomical variability [14]. Consequently, it is mandatory, beforehand, to speak about fascial alterations or dysfunctions in pathological conditions to clearly know the normal aspect of the superficial fascia of the body, codifying the best probe position to visualize them inside the subcutaneous tissue. While this knowledge is present for the deep/muscular fascia [11,14,15], to date, no study has evaluated the superficial fascia thickness of the arm and forearm measured by US imaging in the different regions and levels.

Furthermore, it is well-known from dissection that in the upper limb, the superficial fascia is present in the entire limb [2], showing the differences in the various regions and levels with the naked eye.

The main purpose of this study was to codify the best positions for the probe for studying the superficial fascia of the upper limb and understanding if it has constant features in the various regions and levels and if its thicknesses are different in the different topographical regions. The second aim was to assess the intra-reliability of the US evaluation of the superficial fascia of the upper limb.

## 2. Materials and Methods

### 2.1. Study Design

A cross-sectional study based on the Strengthening Reporting of Observational Study in Epidemiology (STROBE) statement was conducted [16] to compare the US thicknesses of superficial fascia in different compartments and levels of the arm and the forearm. The Helsinki Declaration and human experimentation rules [17] were considered, and the Ethics Committee of University of Padua evaluated the research. All participants were informed prior to inclusion in the project by providing a written consent form.

### 2.2. Participants

A total sample of 30 subject were recruited aged between 20 and 60 years. The participants were excluded if they had any upper extremity injuries (e.g., previous fractures, tendinopathies, tendon ruptures, or neuropathy injuries; past diagnosis of a neuromusculoskeletal condition of arm or forearm, e.g., use of palmar orthoses, carpal tunnel syndrome etc.; or past diagnosis of a neuro-musculoskeletal condition of the arm and forearm, e.g., degeneration or inflammation of the homerus periosteum) or surgery, severe orthopedic, neuronal, psychiatric, cardiopulmonary, or endocrine diseases, were under 18 years old, pregnant, with a chronic skin condition (eczema, psoriasis, lymphedema, lipedema etc.), had previous severe trauma in the inferior limbs, collagen disorder (scleroderma, mixed connective tissue disorder, etc.), and/or chronic medical condition requiring intake of medications. The enrolment of the subjects was performed by a specialized medical doctor with more than 5 years of experience in physical and rehabilitation medicine.

### 2.3. Ultrasonography Imaging Measurements

Using a high-resolution device (Sonosite Edge II, FUJIFILM, Inc. 21919, Bothell, WA, USA) with a 6–15 MHz linear transducer (HLF50x, Sonosite Edge II, FUJIFILM, Inc. 21919, WA, USA) and a screen resolution of 1680 × 1050 pixels, ultrasound images were taken of the arm and forearm with a specific protocol in accordance with Pirri et al. [14]. A physician specialist in physical and rehabilitation medicine with 7 years’ experience in skeletal-muscle US imaging and US imaging of fasciae carried out the US measurements. 

**Figure 1:**
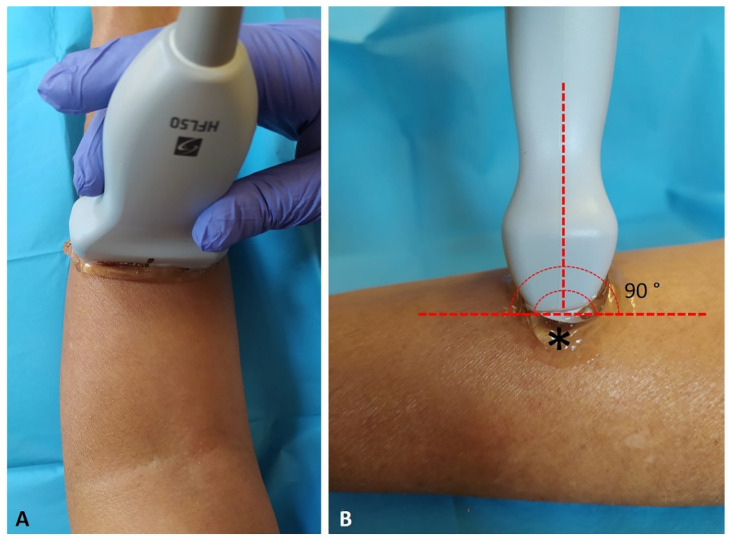
The probe position during the ultrasound (US) imaging assessment of the superficial fascia. (**A**): the anterior region of the forearm (Ant 1); (**B**) for adequate scans and to reduce surface pressure on the skin, the ultra-sonographer used suitable amounts of gel (*), and the US beam was kept perpendicular to the fascial layers.

The US system speed of sound was c = 1540 nm/s, conventional for use in diagnostic US. The US was set to B-mode and depicted a depth of 15 mm. For adequate scans and to reduce surface pressure on the skin, the ultra-sonographer used suitable amounts of gel. The probe was placed on the skin as lightly as possible to avoid tissue compression but was quite stable to maintain adequate contact between the probe and the skin for consistent images. The US beam was kept perpendicular to the fascial layers because anisotropy artifacts typically affect them (Figure 1). The power and overall gain of the US machine were adjusted to optimize visualization of the fascial layers and obtain the best scan possible [14]. The investigator used the short axis in according to Pirri et al. [14].

The US images were frozen, capture, and acquired at the end of each assessment; the superficial fascia thickness was measured using Image J software. To eliminate the influence of possible thickness variations, three equidistant regions of interest per image/level for superficial fascia were measured; in each of them, three points representing the best visibility for each superficial fascia layer were measured, and the resulting values were averaged for analysis. The rater followed the same protocol to ensure that each point of superficial fascia in the arm and in the forearm was quantified in the same way. Moreover, the same procedure of image assessment was performed three different times to calculate the reliability of the measurements.

**Figure 2:**
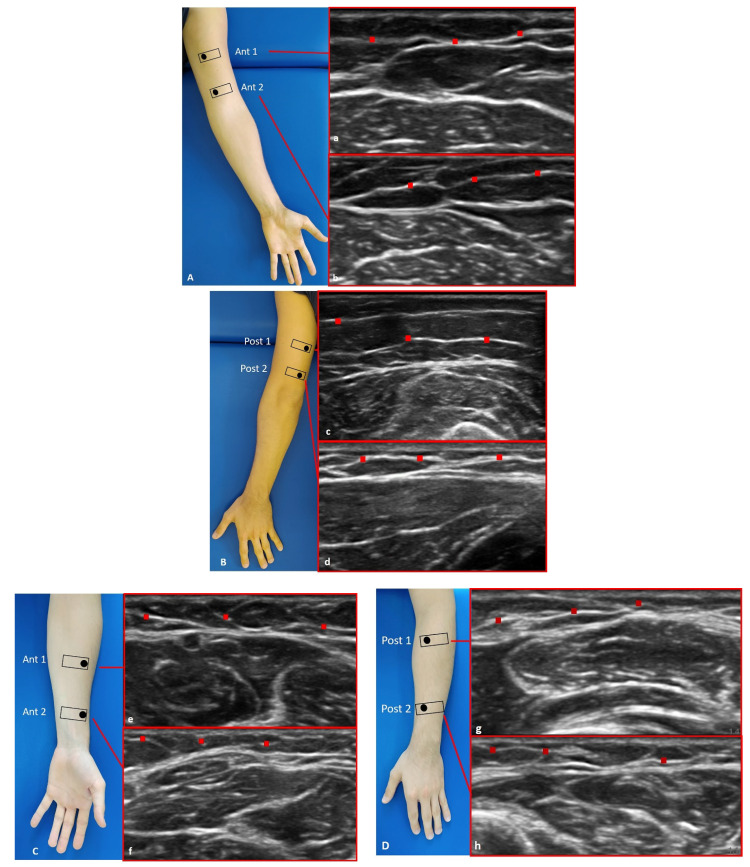
Ultrasound (US) images of the superficial fascia of: the anterior region of the arm (**A**) and of the forearm (**C**); the posterior region of the arm (**B**) and of the forearm (**D**). Anterior regions (**A**,**C**) at levels Ant 1 (**a**,**e**) and Ant 2 (**b**,**f**). Posterior regions (**B**,**D**) at levels Post 1 (**c**,**g**) and Post 2 (**d**,**h**). Probe: black rectangle; Red dashes: superficial fascia.

For each point, we followed the description of the fascial layer visualization in US imaging used by Pirri et al. [11] and followed the protocol by Pirri et al. [14] to capture the US images for arm: anterior region (Ant1 and Ant 2) (Figure 2A(a,b)) and posterior region (Post 1 and Post 2) (Figure 2B(c,d)); for forearm: anterior region (Ant1 and Ant 2) (Figure 2C(e,f)) and posterior region (Post 1 and Post 2) (Figure 2D(g,h)).

### 2.4. Statistical Analysis

Statistical analysis was performed using GraphPad PRISM 8.4.2 (GraphPad Software Inc., San Diego, CA, USA), and a *p* < 0.05 was always considered the limit for statistical significance. The resulting effect size was calculated by G power 3.1 (Universität Düsseldorf: Psychologie) according to Cohen’s d and interpreted as small (d = 0.20), medium (d = 0.50), or large (d = 0.80) [16]. For the superficial fascia of the arm and forearm, the effect size was d = 1.2 in a first our pilot study confirmed from other study [9], α error prob. = 0.05, power: 1-β err prob = 0.95; total sample size was = 10 [18]. Nevertheless, we could include a sample of 30 healthy volunteers in our group.

The normality assessment was carried out using Kolgomorov–Smirnov test. Descriptive statistics were calculated, including measures of central tendency and their dispersion ranges using mean and standard deviation (SD) to describe the parametric data. Differences in US-estimated thickness of the superficial fascia in the arm and in the forearm across regions/levels were statistically analyzed with one-way analysis of variance (ANOVA) followed by Tukey’s test for multiple comparisons. In addition, the Pearson’s test was employed to evaluate the correlations between BMI, weight, height, age, and superficial fascia of arm and forearm.

Moreover, two-way mixed-model intra-class correlation coefficient (ICC2, k), type A, k, was used to evaluate the intra-rater reliability. ICC values were interpreted as poor when below 0.5, as moderate when between 0.5 and 0.75, as good when between 0.75 and 0.90, and as excellent when above 0.90 [19]. SPSS version 21 was used for the analysis of reliability (SPSS Inc., Chicago, IL, USA).

## 3. Results

A total of 30 subjects (16 female and 14 male) participated in this study. The descriptive data of the sample are summarized in Table 1.

### 3.1. Ultrasound Measurements of Superficial Fascia of the Arm

**Figure 3:**
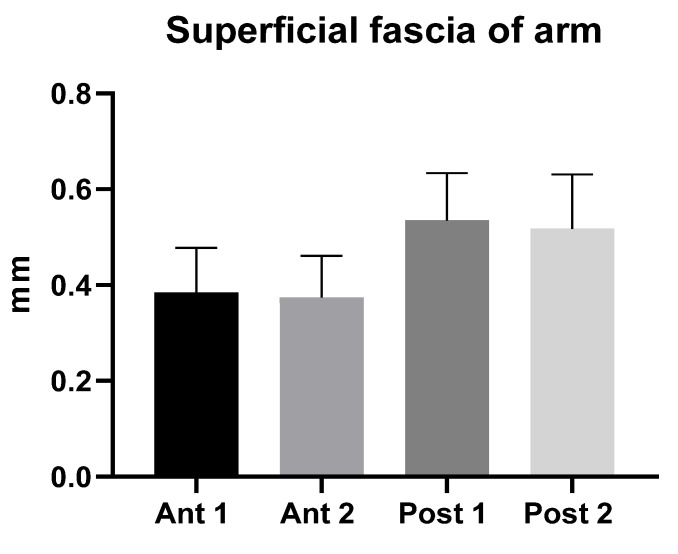
Ultrasound thickness measurements of the superficial fascia of the arm.

The superficial fascia is a fibrous-elastic connective tissue with fat that is mingled in the posterior region at different levels of arm, that can be observed to be double, and that holds the subcutaneous tissue together. The superficial fascia of the arm had a mean US thickness of 0.45 ± 0.10 mm (Table 2 and Figure 3).

The superficial fascia was thicker (*p* < 0.0001) in the posterior region (0.53 ± 0.10 mm) than in the anterior region (0.40 ± 0.10 mm), whilst there was no difference between the proximal and the distal levels (Table 3). Moreover, no differences were found between right and left sides for all regions and levels (*p* > 0.05). The findings for the comparisons within different regions/levels of the superficial fascia are reported in Table 3. According to Tukey’s multiple comparison test, the comparison between superficial fascia thickness among various levels/regions of the arm showed statistically significant differences (Table 3).

### 3.2. Ultrasound Measurements of the Superficial Fascia of Forearm

**Figure 4:**
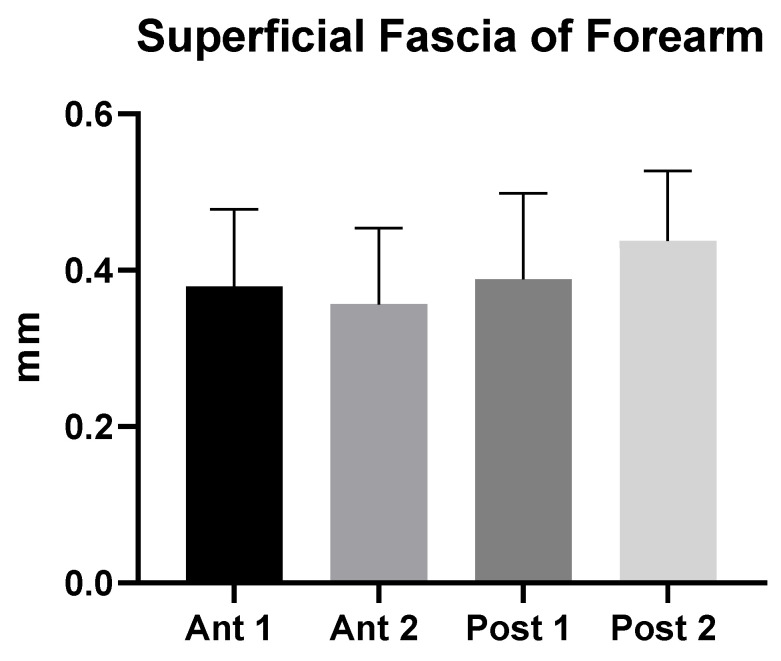
Ultrasound thickness measurements of the superficial fascia of the forearm.

The superficial fascia of the forearm had a mean US thickness of 0.40 ± 0.04 mm (Table 4 and Figure 4).

The superficial fascia had a mean thickness of 0.41 ± 0.1 mm in the posterior region compared with a mean thickness of 0.40 ± 0.10 mm in the anterior region (Table 4). Moreover, no differences were found between right and left sides for all regions and levels (*p* > 0.05). In addition, the comparison within different regions/levels of the superficial fascia of forearm are reported in Table 5. According to Tukey’s multiple comparison test, the comparison between superficial fascia thickness among various levels/regions of the forearm showed some statistically significant difference (Table 5).

### 3.3. Ultrasound Measurements Comparison between the Superficial Fascia of Arm and Forearm

**Figure 5:**
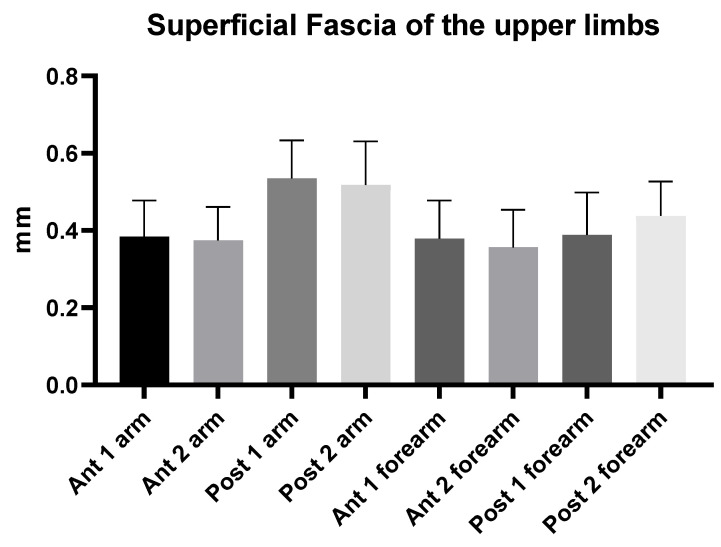
Ultrasound thickness measurements of the superficial fascia of the arm and forearm at different regions/levels.

According to Tukey’s multiple comparison test (Table 6 and Figure 5), the comparisons between different regions/levels of the superficial fascia of arm and of forearm showed statistically significant differences, with an alternating trend between the anterior and posterior region of the superficial fascia of the arm and of forearm (Table 6). Regarding the posterior region/levels, the superficial fascia of the arm had a greater thickness (0.45 ± 0.10 mm) than the superficial fascia of the forearm (0.40 ± 0.10 mm) (Figure 5 and Table 6).

### 3.4. Correlation Ultrasound Measurements and Descriptive Data

#### 3.4.1. Correlation Superficial Fascia of Arm Ultrasound Measurements and Descriptive Data

According to correlation analysis (Table 7), there were statistically significant correlations between superficial fascia thickness and BMI, and the correlation was significant in Anterior 2 level.

#### 3.4.2. Correlation Superficial Fascia of forearm Ultrasound Measurements and Descriptive Data

According to correlation analysis (Table 8), there were statistically significant correlations between superficial fascia thickness and age, BMI, height, weight.

### 3.5. Intra-Rater Reliability

In addition, the intra-rater reliability was good. The results for the superficial fascia of the arm were: anterior region (ICC_2,k_: 0.88; 0.85–0.90), and posterior region(ICC_2,k_: 0.90; 0.85–0.95), and for the superficial fascia of the forearm: anterior region (ICC_2,k_: 0.88; 0.85–0.90), and posterior region (ICC_2,k_: 0.88; 0.85–0.90) (Table 9).

## 4. Discussion

To the current knowledge, this is the first study that thoroughly assessed the US thickness superficial fascia of the upper limbs at different regions and levels.

Prior work has documented and assessed the presence of this structure inside the subcutaneous tissue in another topographical region [20] with US imaging. The superficial fascia was visualized in all regions and levels, appearing as a waving hyperechogenic layer inside the subcutaneous tissue, deep in the (epi)dermis, dividing the subcutaneous tissue in two compartments: the superficial adipose tissue (SAT) and the deep adipose tissue (DAT) [11].

The study’s primary aim was to measure the different superficial fascia thicknesses in different regions and levels of the arm and the forearm among healthy volunteers.

The study’s findings for the arm showed that the superficial fascia in the posterior region at the different levels was thicker (0.53 ± 0.10 mm) than in the anterior region (0.40 ± 0.10 mm) (Table 2), showing a significant statistical difference (*p* < 0.0001) (Table 3 and Figure 2). On the contrary, in the forearm, the superficial fascia showed statistically significant differences between the anterior and the posterior regions only for some levels (Table 5 and Figure 3). Its US mean thickness was 0.41 ± 0.10 mm in the posterior region and 0.40 ± 0.10 mm in the anterior region (Table 4 and Figure 3). It was thicker at Post 2 level, probably because of the proximity to the wrist, where the superficial fascia and deep fascia merge to form the extensor wrist retinacula [21]. Therefore, the differences between the arm and forearm were statistically significant (Table 6) within different regions/levels. Moreover, in some cases, it is mandatory to distinguish the anatomical structures by correct methodology following the protocol and the various techniques used during the ultrasound examination, such as a disto-proximal lift to distinguish, for example, the retinacula cutis/skin ligaments from the fascia, which is the only continuous one between the two structures. Qualitatively, the superficial fascia is homogenous both anteriorly and posteriorly, and this homogeneity was confirmed, also quantitatively, with not significant differences (*p* > 0.05) inside the same region but between the different levels; the superficial fascia was present between regions at significance (*p* < 0.0001). The results of this paper showed that if a qualitative alteration is found during the exams by disto-proximal lift evaluation, a quantitative evaluation must be carried out highlighting differences in the same region that could be the indicator of a hypothetical fascial dysfunction.

These findings indicate that the superficial fascia in the arm and in forearm tends to be thicker posteriorly, indicating a greater role in organizing the subcutaneous tissue in these compartments [20]. Indeed, the superficial fascia, being a fibrous-elastic connective tissue with fat mingled, was observed to be double in the posterior region at different levels of arm, holding the subcutaneous tissue together and allowing it to maintain its integrity in the transfer of forces during the movement [22]. Moreover, the superficial fascia splits around major subcutaneous vessels and nerves [11]. All this increases the variability of its thickness.

These findings are extremely important because they highlight how the superficial fascia and the deep fascia are totally different in terms of not only histological but also ultrasound characteristics, in particular in their thickness. Indeed, as has been reported by other studies examining the deep fascia of the arm and forearm, US thickness was reported to be on average 0.71 ± 0.13 mm for arm and 0.70 ± 0.2 mm for forearm [14], respectively, larger than superficial fascia.

These results confirmed, as has been demonstrated for the deep fasciae [14,15], that there is good or optimal intra- and inter-reliability in the US assessment of the fasciae when the sonographers have fascial anatomy knowledge and optimal technical skills [23,24]. However, using short, axial and transversal scans, the superficial fascia was easily identified in all regions and levels analysed, appearing as linear, laminate or bi-laminate hyper-echoic layers, within the context of the hypo-echogenic subcutaneous adipose tissue. All this was more evident and clearer in the posterior regions/levels, in which the superficial fascia showed the best visibility, but the US assessment in all region/levels is fundamental for a complete evaluation of this structure.

The subcutaneous tissue is a fuel storage unit under the strict control of neuroendocrine system [25]. The WHO defines overweight and obesity as abnormal and excessive fat accumulation in the body, and these are usually classified by body mass index (BMI) [26]. Subcutaneous fat diseases involving adipose tissue and its superficial fascia appear in particular in the spectrum of obesity [27,28], and in this preliminary study, an analysis of study correlation results showed that US measurements had relationships with the descriptive data (age, height, weight, and BMI). Indeed, in the arm, US superficial fascia thickness showed relationships at the Ant 2 level with BMI and weight (Table 7), while in the forearm, the relationships were statistically significant with age, BMI, height, and weight (Table 8). This correlation can be explained by the fact that the superficial fascia has a crucial role in the holding, organization, and storage of adipose tissue, increasing its thickness to sustain the adipose tissue [29].

An association of forearm superficial fascia thickness and age was found at level Ant 2 with a positive correlation. We hypothesize that this correlation is due to aging, in a topographical region in which the superficial fascia and deep fascia start to merge to form the retinacula of wrist. Loads and various patterns of movement age the superficial fascia [30,31].

The importance of superficial structure in the composition of subcutaneous tissue suggests the use of US imaging for also evaluating the superficial fascia thickness in the case of subcutaneous pathologies to have another diagnostic parameter because US is an inexpensive, safe, portable, and most of all, effective tool [11,32]. Moreover, because the superficial fascia is a continuum inside the subcutaneous tissue, the difference between the anterior and posterior regions is fundamental information during the ultrasound examination; any thickness increase in the anterior vs. posterior regions could raise the question of a possible dysfunction and needs to be associated with the patient’s clinic/symptomatology [11]. Therefore, the differences between forearm and arm must be contextualized since many pathologies of the subcutaneous tissue could alter these thicknesses and deserve to be measured. Many subcutaneous adipose tissues diseases [33,34,35] have fat within the subcutaneous tissue that grows abnormally in amount or structure, often causing pain and other discomfort. For example, obesity is a main cause of the densification and fibrosis of superficial fascia, forming a fibrotic mesh around adipocytes and fat lobules [36,37]. In the future, pathologies such as lymphedema and lipoedema, characterized by fibrosis of the subcutaneous tissue [33,34,35] and never previously evaluated, could benefit from a better staging that takes into account the thickness of the superficial fascia. Having parameter values in healthy subjects will allow for the better evaluation of patients in various pathological situations.

This is the first work to our knowledge to examine and compare the thicknesses of the superficial fascia in the arm and forearm using US imaging. Future longitudinal studies including not only healthy volunteers but also a large population of patients will contribute to our knowledge of the pathophysiology of different thicknesses. Finally, being able to assess this structure involved in fascial dysfunctions would help to target the treatment of these structures.

### Limitations of the Study

The small number of healthy volunteers included in this study cohort and the qualitative aspect of the assessments mean that it is not possible to statistically analyse the prevalence of US findings or to explain their possible causes, prognostic significance, and therapeutic implications. Finally, US evaluation of superficial fascia morphology greatly depends on the knowledge and skills of the investigator as well as the proper setting of the device.

## 5. Conclusions

US refines visual evaluation of the superficial fascia. In addition, it may reveal changes not highlighted on normal clinical inspection. A few of these changes require further investigation because they have not yet been explained or described. Accordingly, US may help to improve the grading of superficial fascial dysfunction or disease by revealing subclinical lesions and clinically invisible fascial changes.

## Figures and Tables

**Table 1:** Descriptive data of the sample.

Descriptive Statistics	Age	BMI	Height	Weight
Number of values	30	30	30	30
Minimum	20	15.79	158	43
Maximum	60	31.6	183	87
Range	40	15.81	25	44
Mean	33.23	23.27	170.7	67.28
Std. Deviation	13.31	3.692	6.865	13.54
Coefficient of variation	40.06%	15.86%	4.022%	20.12%

**Table 2:** Ultrasound thickness measurements of the superficial fascia of the arm.

Descriptive Statistics	Ant 1	Ant 2	Post 1	Post 2
Number of values	60	60	60	60
Minimum	0.30	0.31	0.34	0.40
Maximum	0.62	0.51	0.75	0.82
Range	0.32	0.20	0.41	0.42
Mean	0.40	0.40	0.54	0.52
Std. Deviation	0.10	0.10	0.10	0.11
Std. Error of Mean	0.01	0.01	0.01	0.01
Coefficient of variation	24.40%	23.10%	18.50%	21.90%

**Table 3:** Ultrasound measurements within different regions/levels of the superficial fascia of the arm. Statistically significant results are showed in bold. ****: *p* < 0.0001. ns: not statistically significant.

Type of Comparison	Mean Diff.	95.00% CI of Diff.	Significant?	Summary	Adjusted *p* Value
Ant 1 vs. Ant 2	0.0098	−0.0366 to 0.0563	No	ns	0.9472
Ant 1 vs. Post 1	−0.1508	−0.1973 to −0.1043	**Yes**	****	<0.0001
Ant 1 vs. Post 2	−0.1337	−0.1802 to −0.0871	**Yes**	****	<0.0001
Ant 2 vs. Post 1	−0.1607	−0.2072 to −0.1142	**Yes**	****	<0.0001
Ant 2 vs. Post 2	−0.1435	−0.1900 to −0.0970	**Yes**	****	<0.0001
Post 1 vs. Post 2	0.0171	−0.0293 to 0.0636	No	ns	0.7749

**Table 4:** Ultrasound thickness measurements of the superficial fascia of the forearm.

Descriptive Statistics	Ant 1	Ant 2	Post 1	Post 2
Number of values	60	60	60	60
Minimum	0.30	0.30	0.37	0.35
Maximum	0.64	0.60	0.72	0.71
Range	0.34	0.30	0.35	0.36
Mean	0.38	0.36	0.40	0.44
Std. Deviation	0.10	0.10	0.11	0.10
Std. Error of Mean	0.01	0.01	0.01	0.01
Coefficient of variation	26.08%	27.28%	28.30%	20.42%

**Table 5:** Ultrasound measurements within different regions/levels of the superficial fascia of the forearm. Statistically significant results are showed in bold. *: *p* < 0.05; **: *p* < 0.01; ****: *p* < 0.0001. ns: not statistically significant.

Type of Comparison	Mean Diff.	95.00% CI of Diff.	Significant?	Summary	Adjusted *p* Value
Ant 1 vs. Ant 2	0.0223	−0.0244 to 0.0691	No	ns	0.6055
Ant 1 vs. Post 1	−0.0093	−0.0561 to 0.0374	No	ns	0.9552
Ant 1 vs. Post 2	−0.0586	−0.1055 to −0.0118	**Yes**	**	0.0074
Ant 2 vs. Post 1	−0.0316	−0.0784 to 0.0151	No	ns	0.3001
Ant 2 vs. Post 2	−0.0810	−0.1278 to −0.0341	**Yes**	****	<0.0001
Post 1 vs. Post 2	−0.0493	−0.0961 to −0.0025	**Yes**	*	0.0344

**Table 6:** Ultrasound measurements within different regions/levels of the superficial fascia of arm and of forearm. Statistically significant results are showed in bold. *: *p* < 0.05; ***: *p* < 0.001; ****: *p* < 0.0001. ns: not statistically significant.

Type of Comparison	Mean Diff.	95.00% CI of Diff.	Significant?	Summary	Adjusted *p* Value
Ant 1 arm vs. Ant 1 forearm	0.0053	−0.0495 to 0.0602	No	ns	>0.9999
Ant 1 arm vs. Ant 2 forearm	0.0276	−0.0272 to 0.0825	No	ns	0.7885
Ant 1 arm vs. Post 1 forearm	−0.004	−0.0589 to 0.0509	No	ns	>0.9999
Ant 1 arm vs. Post 2 forearm	−0.0533	−0.1082 to 0.0015	No	ns	0.0639
Ant 2 arm vs. Ant 1 forearm	−0.0045	−0.0594 to 0.0504	No	ns	>0.9999
Ant 2 arm vs. Ant 2 forearm	0.0178	−0.0370 to 0.0727	No	ns	0.9759
Ant 2 arm vs. Post 1 forearm	−0.0138	−0.0687 to 0.0410	No	ns	0.9946
**Ant 2 arm vs. Post 2 forearm**	**−0.0631**	**−0.1181 to −0.0082**	**Yes**	*	**0.0117**
**Post 1 arm vs. Ant 1 forearm**	**0.1562**	**0.1013 to 0.2111**	**Yes**	****	**<0.0001**
**Post 1 arm vs. Ant 2 forearm**	**0.1785**	**0.1236 to 0.2334**	**Yes**	****	**<0.0001**
**Post 1 arm vs. Post 1 forearm**	**0.1468**	**0.0919 to 0.2017**	**Yes**	****	**<0.0001**
**Post 1 arm vs. Post 2 forearm**	**0.0975**	**0.0426 to 0.1524**	**Yes**	****	**<0.0001**
**Post 2 arm vs. Ant 1 forearm**	**0.139**	**0.0841 to 0.1939**	**Yes**	****	**<0.0001**
**Post 2 arm vs. Ant 2 forearm**	**0.1613**	**0.1064 to 0.2162**	**Yes**	****	**<0.0001**
**Post 2 arm vs. Post 1 forearm**	**0.1297**	**0.0747 to 0.1846**	**Yes**	****	**<0.0001**
**Post 2 arm vs. Post 2 forearm**	**0.0803**	**0.0254 to 0.1352**	**Yes**	***	**0.0003**

**Table 7:** Correlations (Pearson R coefficient test) between the superficial fascia of the arm: Ultrasound measurements and descriptive data. Only statistically significant data are reported. BMI = body mass index.

Type of Region/Level	Data	r	*p*-Value	95%CI of Diff.
Ant 2	BMI	0.3688	0.0037	0.1267 to 0.5694
Ant 2	Weight	0.3792	0.0388	0.0219 to 0.6506

**Table 8:** Correlation (Pearson R coefficient test) between the superficial fascia of the arm ultrasound measurements and the descriptive data. Only statistically significant data are reported.

Type of Region/Level	Data	r	*p*-Value	95%CI of Diff.
Ant 2	Age	0.2823	0.0289	0.0305 to 0.5003
Ant 1	BMI	0.3062	0.0173	0.0567 to 0.5198
Ant 2	BMI	0.2755	0.0331	0.0232 to 0.4948
Post 1	BMI	0.3163	0.0138	0.0677 to 0.5278
Ant 1	Height	0.2729	0.0349	0.0203 to 0.4927
Post 1	Weight	0.2584	0.0462	0.0047 to 0.4808

Abbreviations: BMI = body mass index.

**Table 9:** Intra-rater reliability of the ultrasound measurements within different regions/levels of the superficial fascia of the arm and of forearm. Sup.: superficial.

Type of Fascia	Region	ICC
Sup. Fascia arm	Anterior	0.88 (0.85–0.90)
Sup. Fascia arm	Posterior	0.90 (0.85–0.95)
Sup. Fascia forearm	Anterior	0.88 (0.85–0.90)
Sup. Fascia forearm	Posterior	0.88 (0.85–0.90)

## Data Availability

The data presented in this study are available on request from the corresponding author. The data are not publicly available due to privacy.
